# Debate: Where to next for universal school‐based mental health interventions? Time to move towards more effective alternatives

**DOI:** 10.1111/camh.12753

**Published:** 2024-12-07

**Authors:** Jack L. Andrews, Lucy Foulkes

**Affiliations:** ^1^ Department of Experimental Psychology University of Oxford Oxford UK

**Keywords:** Prevention, Intervention, school, mental health

## Abstract

There is an urgent need to improve mental health outcomes among young people. One approach taken to address this problem has been the design and delivery of universal school‐based prevention, based on therapeutic models such as CBT and mindfulness. Such interventions are delivered to groups of young people, irrespective of risk or need. However, in this commentary, we argue that the initial appeal of universal interventions has not been supported by the evidence: universal school‐based prevention is less effective than targeted approaches, often leads to null or unsustained positive effects, has the potential to elicit negative effects and is not well liked by young people themselves. In addition, many young people in each classroom already meet the criteria for a mental disorder, meaning that prevention approaches may not be appropriate or effective for this group. In this commentary, we respond to Birrell et al.'s (2025) paper by arguing that the field should move away from universal prevention and instead invest our limited resources in the refinement and dissemination of interventions with a stronger evidence base, such as one‐to‐one, targeted and indirect approaches.

## Introduction

Universal school‐based prevention has been a widely implemented approach to reduce the onset and progression of mental health problems among young people. These interventions are delivered in groups to all young people in a class or school irrespective of risk or need and are more wide‐reaching, less stigmatising and often easier to implement than one‐to‐one or targeted interventions. In this commentary, we argue that the initial appeal of universal interventions for preventing mental health problems has not been supported by the evidence. We focus on therapeutically informed universal interventions, in which young people learn psychoeducational concepts and practical exercises that are taken from therapeutic modalities such as mindfulness, cognitive behavioural therapy (CBT) or dialectical behavioural therapy (DBT).

There is now evidence from several large‐scale trials that therapeutically informed universal interventions are less effective than targeted approaches (Werner‐Seidler et al., 2021), often lead to null effects or unsustained positive effects and also have the potential to elicit negative effects (Andrews et al., 2022; Montero‐Marin et al., 2022). We agree with Birrell et al. (2025) that the field is at an important reflection point for universal interventions, but it is our position that, given the evidence, investment in school‐based interventions should move away from therapeutically informed universal prevention. Here we present three reasons why the field should divest from these universal interventions and invest more in one‐to‐one, targeted and indirect interventions instead.

### True universal prevention is not possible because too many young people are already symptomatic

In the broader literature, ‘prevention’ can mean a number of different things: interventions that prevent the onset of a disorder before any symptoms appear (*primary prevention*), those that slow or reduce the progression of a disorder when symptoms have already begun (*secondary prevention*) or those that manage or lessen the impact of a fully developed disorder (*tertiary prevention*). Each of these approaches is valid, but they should not be confused with each other, and their effects should not be analysed together. In the school‐based mental health intervention literature, universal preventive interventions are often described as, and intended to act as, primary prevention. However, a sizeable minority of young people in every class will already meet the criterita for a mental disorder: the estimated prevalence of common mental disorders among adolescents is 25 ‐ 31% (Silva et al., 2020). An even larger percentage will show elevated symptoms. This means that, within any one classroom, a supposed primary prevention intervention is being delivered to three groups of young people: those with no symptoms at baseline, those who are symptomatic but do not meet the threshold for disorder and those who have a clinical disorder. Only the first group is receiving a true (primary) prevention intervention. To assess an intervention's true preventative effects, outcomes should be analysed only among this group, but this analysis is not typically reported (Fazel & Kohrt, 2019).

This is not merely an issue of semantics or statistical analysis. Delivering a ‘preventative’ one‐size‐fits‐all intervention to individuals who are already symptomatic may be actively problematic. First, it may exacerbate symptoms. Studies have shown that both mindfulness‐ and CBT‐based universal interventions have led to an increase in mental health symptoms among individuals with pre‐existing symptoms (see below; Montero‐Marin et al., 2022; Stallard et al., 2013). It may be that the taught strategies were irrelevant or inadequate, leading to increased distress. There is qualitative evidence that some young people find school universal interventions irrelevant to them or the skills presented too difficult to enact (Bailey et al., 2023; Miller, Crane, Medlicott, Robson, & Taylor, 2023), creating an opportunity cost: some individuals spend time on an intervention that is inadequate for them when they could be engaging in more appropriate support (Foulkes, Andrews, Reardon, & Stringaris, 2024).

### Universal therapeutically informed prevention can have negative effects

Iatrogenic effects among universal school prevention have been documented, with some individuals faring worse following the intervention, relative to the control group. In a recent scoping review of school‐based group mental health interventions, we found that among high‐quality studies (i.e. those with low risk of bias), a third (33.33%) reported at least one negative outcome (Guzman Holst, Davis, Andrews, & Foulkes, 2024). This included an increase in internalising symptoms, a decrease in prosocial behaviour and a decrease in parental relationship quality. Around half of these negative outcomes were found within subgroup analyses: for example, being younger, experiencing deprivation or having mental health problems at baseline were all associated with worse outcomes (Guzman Holst et al., 2024).

Even when there are no negative effects, universal interventions are likely not potent enough to bring about meaningful improvement in young people's mental health. For example, one systematic review of school‐based mental health interventions found that effect sizes for long‐term (>12 months) follow‐ups of universal interventions were trivial (i.e. <.1; *g* = 0.07 for depression and *g* = 0.09 for anxiety; Werner‐Seidler et al., 2021). In addition, these effect sizes should be understood within the context of ‘voltage drop’, whereby intervention effects often become further diluted outside of trial contexts (Chambers, Glasgow, & Stange, 2013).

The findings regarding negative effects alone are not necessarily a reason to move away from universal interventions, but the possibility of negative effects in the context of null effects and small, temporary positive effects (that might be further diluted in the real world) signals the need for a different approach. All interventions have the capacity to have a negative impact at least some of the time; this is accepted and tolerated if there is a cost–benefit ratio that suggests the intervention is still worth doing (Foulkes et al., 2024). We argue that, for universal therapeutically informed school mental health interventions, the potential positive effects are not large enough nor sustained for long enough to warrant their delivery, given the known potential for these interventions to lead to null or negative effects.

### Universal prevention is not well accepted and does not give young people autonomy and choice

For a universal intervention to be effective, it must be acceptable to the target population: that is, young people must like the intervention and feel that it meets their needs. However, a number of recent trials have shown poor acceptability among adolescents. For example, follow‐up data from a CBT‐based trial found that less than half (49%) of participants thought that the content was ‘completely’ or ‘somewhat relevant’ to their lives, and only 29% would recommend the program to their friends (Bailey et al., 2023). Poor outcomes related to student engagement were also observed in the MYRIAD mindfulness trial (Miller et al., 2023).

The extent to which it is feasible and cost‐effective to keep refining universal interventions should be considered. Given the sizeable individual differences among young people in any one classroom (with regard to mental health problems, neurodiversity, gender, culture, SES, etc), it may be impossible to design an intervention that will be liked and perceived as relevant by everyone. Indeed, the universality itself might be the problem: by their very nature, universal interventions do not offer choice to adolescents, yet fostering a sense of agency or autonomy is a key developmental goal of adolescence and something that young people want in interventions. On top of this, as highlighted above, many adolescents do not have mental health problems and do not go on to develop one across the period of assessment: we found that 57% of young people in the MYRIAD trial followed a trajectory of stable low mental health problems from the beginning to the end of the intervention, and at follow‐up. It is thus legitimate that many do not find a mental health intervention relevant to them. Instead of seeing low acceptability as a challenge to be overcome (Birrell et al., 2025), we might consider the alternative that these results present us with evidence that the majority of young people do not enjoy or want universal prevention, and that this is a crucial finding we should listen to and respect.

## Where to next?

In this commentary, we have presented three reasons why we should move away from therapeutically informed universal school‐based mental health interventions. While Birrell et al. advocate for continued investment in universal prevention, several of their recommendations  suggest a move away from the current model (e.g. they advocate for a focus on tailored interventions or those which use a branching logic towards targeted activities). In turn, we argue that the field should move towards alternative school‐based approaches with a stronger evidence base, including (1) targeted interventions that focus on smaller groups of individuals at risk of specific problems, (2) opt‐in interventions which are more in line with adolescents' desire for autonomy, (3) indirect interventions which focus on adjacent risk factors such bullying and (4) approaches which increase access to treatment outside of school. One such intervention that meets these specifications is the recent BESST trial, in which adolescents self‐referred to a school‐based group CBT workshop for depressive symptoms (Brown et al., 2024). The authors reported, on average, clinical improvement (*d* = .17) in the intervention group, relative to treatment as usual (Brown et al., 2024). In summary, and in the context of rising rates of mental health problems among young people, we argue that the field should focus more on improving and disseminating effective targeted interventions that have a higher likelihood of improving the mental health of young people.

## Conflict of interest statement

The authors declare no conflicts of interest.

## Author contributions

JLA and LF contributed equally.

## Funding information

JLA is funded by Wellcome (WT/227640/Z/23/Z) and LF is funded by the Prudence Trust (CQR02370).

## Ethical information

No ethical approval was required for this article.

## Data Availability

Data sharing not applicable to this article as no datasets were generated or analysed during the current study.
